# Mesenchymal stem/stromal cell-derived exosomes in regenerative medicine and cancer; overview of development, challenges, and opportunities

**DOI:** 10.1186/s13287-021-02378-7

**Published:** 2021-05-21

**Authors:** Ali Hassanzadeh, Heshu Sulaiman Rahman, Alexander Markov, Judi Januadi Endjun, Angelina Olegovna Zekiy, Max Stanley Chartrand, Nasrin Beheshtkhoo, Mohammad Amin Jadidi Kouhbanani, Faroogh Marofi, Marzieh Nikoo, Mostafa Jarahian

**Affiliations:** 1 Department of Applied Cell Sciences, School of Advanced Technologies in Medicine, Tehran University of Medical Sciences, Tehran, Iran; 2 Department of Physiology, College of Medicine, University of Suleimanyah, Sulaymaniyah, Iraq; 3 Tyumen State Medical University, Tyumen, Russian Federation; 4 Medical Faculty, UPN Veteran, Jakarta, Indonesia; 5 Gatot Soebroto Indonesia Army Hospital, Jakarta, Indonesia; 6 Sechenov First Moscow State Medical University, Moscow, Russia; 7 DigiCare Behavioral Research, Casa Grande, AZ USA; 8 Department of Medical Nanotechnology, School of Advanced Medical Sciences and Technologies, Shiraz University of Medical Sciences, Shiraz, Iran; 9 Immunology Research Center (IRC), Tabriz University of Medical Sciences, Tabriz, Iran; 10 Department of Immunology, School of Medicine, Kermanshah University of Medical Sciences, Kermanshah, Iran; 11 Toxicology and Chemotherapy Unit (G401), German Cancer Research Center, 69120 Heidelberg, Germany

**Keywords:** Mesenchymal stem/stromal cells (MSCs), Exosomes, Regenerative medicine, Cancer, MicroRNAs (miRNAs)

## Abstract

Recently, mesenchymal stem/stromal cells (MSCs) and their widespread biomedical applications have attracted great consideration from the scientific community around the world. However, reports have shown that the main populations of the transplanted MSCs are trapped in the liver, spleen, and lung upon administration, highlighting the importance of the development of cell-free therapies. Concerning rising evidence suggesting that the beneficial effects of MSC therapy are closely linked to MSC-released components, predominantly MSC-derived exosomes, the development of an MSC-based cell-free approach is of paramount importance. The exosomes are nano-sized (30100nm) lipid bilayer membrane vesicles, which are typically released by MSCs and are found in different body fluids. They include various bioactive molecules, such as messenger RNA (mRNA), microRNAs, proteins, and bioactive lipids, thus showing pronounced therapeutic competence for tissues recovery through the maintenance of their endogenous stem cells, the enhancement of regenerative phenotypic traits, inhibition of apoptosis concomitant with immune modulation, and stimulation of the angiogenesis. Conversely, the specific roles of MSC exosomes in the treatment of various tumors remain challenging. The development and clinical application of novel MSC-based cell-free strategies can be supported by better understanding their mechanisms, classifying the subpopulation of exosomes, enhancing the conditions of cell culture and isolation, and increasing the production of exosomes along with engineering exosomes to deliver drugs and therapeutic molecules to the target sites. In the current review, we deliver a brief overview of MSC-derived exosome biogenesis, composition, and isolation methods and discuss recent investigation regarding the therapeutic potential of MSC exosomes in regenerative medicine accompanied by their double-edged sword role in cancer.

## Introduction

**Fig. 1:**
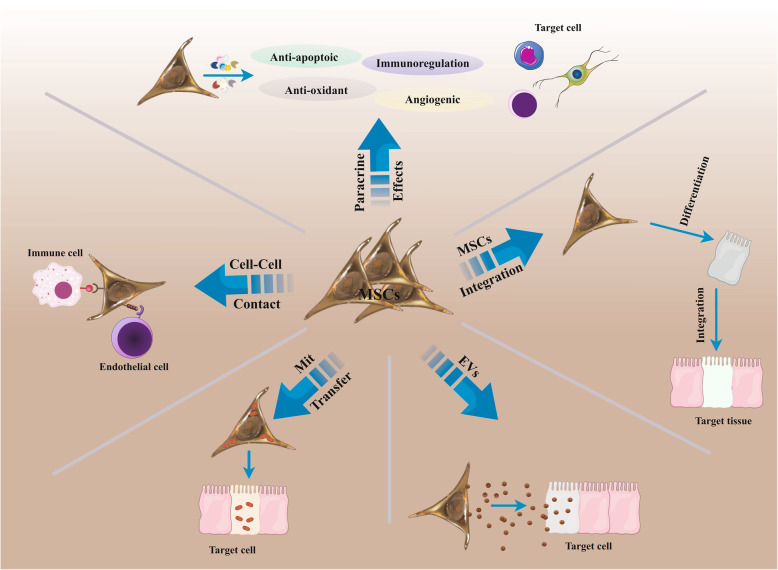
The corresponding mechanisms of MSC-based therapy. MSCs can stimulate restoration in the damaged tissue by differentiation into adult cell lineages and by modifying immune reactions. The immunoregulatory capability of MSCs contains paracrine activity, cell-cell contact, and interaction, mitochondrial (mito) transfer, and secretion of extracellular vesicles (EVs). MSCs; mesenchymal stem/stromal cells

Mesenchymal stem/stromal cells (MSCs) are unique cell populations showing a large potential for unrestricted self-renewal, differentiating into a wide variety of diverse cell lines and forming distinct colonies [1,3]. They are multipotent fibroblast-like stem cells typically found in a variety of human tissue, including bone marrow (BM), adipose tissue, liver, intestine, lung, connective muscle tissue, spleen, skin, placenta, umbilical cords, and other tissues [4,6]. Currently, excellent progress has been established in stem cell technology with promising therapeutic possibilities for the treatment of various diseases [7]. Remarkably, MSCs unique attributes, including differentiation into multiple tissue types, the ability to recruit into damaged sites and tumors, easy isolation process from various tissues, the ability of ex vivo manipulation, and lower ethical concerns [8,10] describe them as an effective therapeutic strategy to induce tissue restoration. Now, considerable efforts have been made to use the MSCs natural interesting capability for homing to inflammation areas, such as those existing in cancer [11, 12]. Meanwhile, another attractive property of MSCs is their lower immunogenicity due to the absence of expressing any co-stimulatory molecules, thus indicating that there is no requirement for the use of immunosuppressive agents for allogeneic transplantation [13, 14]. Based on the literature, they can affect target cells and tissue by cell-cell contact, induction of paracrine effect, the release of exosomes and other secretions, and mitochondria (mito) transfer accompanied by differentiation into a specific adult cell and integration into a target tissue, thus delivering a new platform for cellular and cell-free stem cell therapy (Fig.1) [15, 16]. There is increasing evidence implying that the therapeutic functions and immunoregulatory activities of transplanted MSCs depend extensively on paracrine rather than cellular factors [17, 18]. These studies have shown that most of the transplanted MSCs are commonly trapped in the liver, spleen, and lung, and only less than 1% of MSCs reach and replace at the injured site, emphasizing the prominence of the development of cell-free therapies [19]. The MSCs not only secrete soluble factors (e.g., growth factor, cytokine, chemokine) but also generate and release extracellular vesicles (EVs) which enable eliciting of the therapeutic effects via the exchange of cytoplasmic and genetic material [20, 21]. Moreover, these vesicles resolve safety challenges such as gene mutation and unregulated cell division, and immune rejection commonly is shown during cell-based therapy [15, 22]. In these EVs, exosomes are prominently involved in cell communication and immunomodulatory activities [17]. They are nano-sized (30100nm) lipid bilayer membrane vesicles secreted by MSCs and detected in different body fluids [23,25]. Exosomes may transfer an enormous amount of bioactive molecules to nearby damaged recipient cells, which in turn, cause phenotypic change and then modulate regenerative programs of various organs [26, 27]. These phenotypic changes arise from several mechanisms, ranging from prevention of apoptosis in recipient cells, induction of target cell proliferation, stimulation of immunomodulatory responses, and reduction of oxidative stress in target cells to the enhancement of oxygen supply [28]. Regardless of the role of MSCs in tissue recovery, there is an inconsistency respecting the supportive or suppressive role of MSCs in tumorigenesis or tumor regression [29, 30].

Herein, we discuss MSC-derived exosomes biogenesis and composition and also review the recent investigation concerning the therapeutic potential of MSC exosome in regenerative medicine and their dual role in cancer.

## Exosome biogenesis

**Fig. 2:**
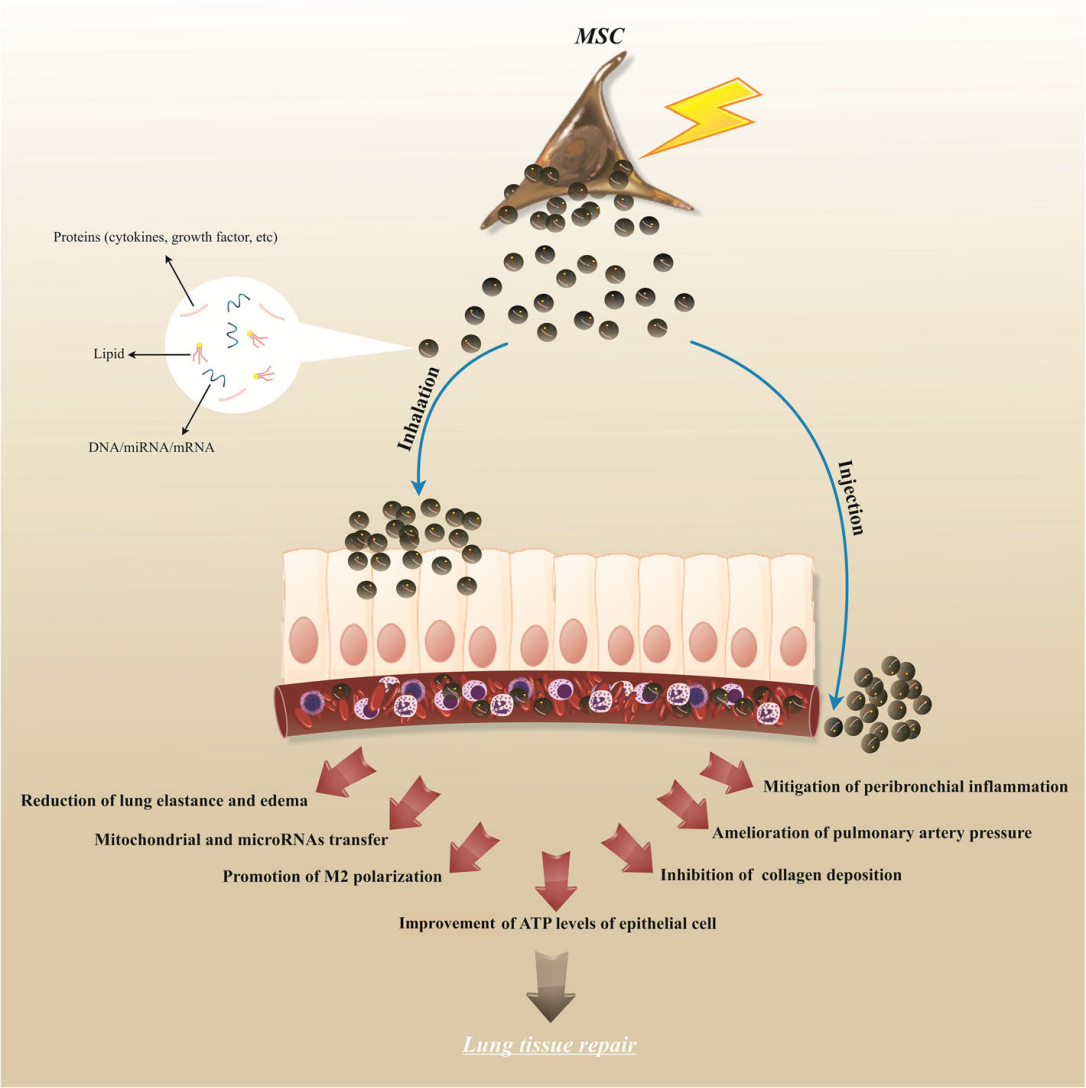
The therapeutic capacity of MSC-derived exosomes in lung disorders. The MSC exosomes could elicit desired therapeutic outcomes in transplanted patients upon administration by intravenous (IV) or inhalation route possibly mediated by their anti-inflammatory, immunomodulatory, pro-angiogenic, and also anti-fibrotic properties. MSCs; mesenchymal stem/stromal cells

Extracellular vesicles (EVs) are lipid bilayer-delimited particles identified as a novel mediator of intercellular communication [31, 32]. They have been categorized according to their size, content, and mechanism of creation over the last decade [33]. There exist three well-known subtypes of EVs, including microvesicles (MVs), apoptotic bodies, and exosomes. The MVs are 100nm^1^m in diameter and are released via direct budding of plasma membrane cells. The other subtype of the EVs are exosomes, 30100nm in diameter, which is generated by inward budding of the intracellular endosomal membrane following the formation of multivesicular bodies (MVBs) (Fig.2) [34, 35]. Finally, apoptotic bodies range from 50 to 5000nm in diameter and commonly are derived from apoptotic cells. The exosomes are frequently secreted by a variety of cell types in culture, including epithelial cells, stem cells, immune cells, and tumor cells. Also, they are commonly present in various biological fluids, such as blood, saliva, urine, and breast milk [36]. Exosome biogenesis and secretion is a complex and unique process that is influenced by cellular origins and intracellular homeostasis, as well as the cells microenvironment and external stimuli [37]. Regarding recent studies, Wnt and mTOR pathways, which increase -catenin expression, are introduced as master regulators required both for MSC exosome secretion and also supporting the self-renewal of MSCs [38,40]. Furthermore, various physiological or pathological conditions can influence the mechanism of biomolecule packing into MSC exosomes as well as their biological function. For example, exosomes derived from hypoxia-exposed MSCs contain higher rates of the angiogenic proteins, such as platelet-derived growth factor (PDGF), fibroblast growth factor (FGF), and epidermal growth factor (EGF), and exert more angiogenic activity than normoxia-exposed MSC-derived exosomes [41, 42]. The mechanism of exosome production during exosome biogenesis includes initiation; the plasma membrane is internalized to form an endocytic vesicle termed an early endosome, followed by the early endosome developments into the late endosome. The budding of late endosomal membranes leads to the development of intraluminal vesicles (ILVs) within large MVBs [43]. Most ILVs are secreted into the extracellular space following merging with the plasma membrane, termed exosomes [44, 45]. In detail, exosome biogenesis is regulated by two separate molecular mechanisms, including endosomal sorting complex required for transport (ESCRT) machinery-dependent and ESCRT-independent. Considering literature, each ESCRT machine complex includes several components. Meanwhile, the ESCRT-0 complex recognizes ubiquitinated cytosolic domains of the transmembrane protein and sorts them into the endosomal membrane [46, 47]. ESCRT-I, II complexes bind to the outer part of the endosomal membrane and induce direct intraluminal buds to form MVBs; on the other hand, the ESCRT-III complex is assembled on the outer surface of the endosomal membrane and facilitates the release of new ILVs from the endosomal membrane during the MVB generation process. Recent evidence also indicates that lipids such as sphingosine-1-phosphate, ceramides, sphingolipids, heat-shock proteins, and tetraspanin proteins (e.g., CD81, CD82, and CD9) are involved in the sorting of cellular material in MVBs and the regulation of exosome release in ESCRT-independent pathways. Therefore, both ESCRT-dependent and ESCRT-independent pathways are synergistically critical for exosome biogenesis and function [33, 48,51]. Interestingly, the formation, content, and quantity of secreted exosomes will depend on whether the cells are exposed to various stress factors or external stimuli. Importantly, under various conditions, exosomes derived from the same cells can contain distinct components [32, 52]. The secreted exosomes are guided and taken up by recipient cells residing in the microenvironment or transported to distant sites by biological fluids [53]. Exosomes are taken up by the target cell through direct binding to the plasma membrane, by ligand-receptor interaction, or via phagocytosis [54,56]. Exosomes from different original cell types contain a particular collection of molecules that are required for their biogenesis and functional properties. MSC-derived exosomes, in particular, not only contain the common tetraspanin proteins CD63, CD9, and CD81, which are involved in the development of intraluminal buds, but also include CD29, CD73, CD90, CD44, and CD105, which are MSC-specific and play an influential role in exosome biogenesis [57].

## Components of MSC-derived exosomes

The molecular composition of exosomes depends substantially on the types of donor cells, epigenetic changes, and various physiological and pathological environmental conditions [54, 58]. The exosomes are highly enriched in various components, including lipids, proteins, nucleic acids, and other components. The lipid component of exosomes encompasses cholesterol, sphingomyelin, hexosyl ceramides, phosphatidylserine, and prostaglandins, enabling exosomes key functions and activities such as exosome rigidity, exosomal endocytosis, membrane trafficking, and signaling [59, 60]. MSC exosomes also contain proteins, mRNAs, and microRNAs (miRNAs), which are delivered to recipient cells and can alter the behavior of adjacent cells. Indeed, MSC exosomes contribute to cellular processes such as transcription, proliferation, adhesion, migration, and differentiation [61]. Furthermore, MSC exosomes are involved in the induction of angiogenesis, inhibition of fibrosis, enhancing of the neuronal survival and differentiation, stimulation of extracellular matrix remodeling, abrogation of local inflammation response, and also the regulation of immune cells activities [42]. Proteomic analyses have revealed more than 4000 different proteins within exosomes playing multifaceted functions [62]. Exosomes protein components include tetraspanins (CD9, CD63, CD81, CD82), which are localized on the exosome surface and participate in cellular interactions, exosome biogenesis components (e.g., ESCRT complex, ALG2-interacting protein X (ALIX), thiazide-sensitive Na-Cl cotransporter (TSC101) and syntenin protein, membrane transport) and membrane fusion proteins (e.g., Ras associated with diabetes (Rab) GTPase and annexins), and also heat-shock proteins (HSP70) [63,65]. Exosome protein components can participate in cancer treatment and also play critical functional roles in the repair of human damaged tissue [42, 66]. It has been documented that PDGF-D derived from the umbilical cord (UC)-MSC-EVs could support the recovery process of infarcted heart cells [67]. Furthermore, it has been shown that the connection of the proinflammatory cytokine CCL2 with its receptor expressed on BM-MSC-EVs could reduce inflammation and support improvement of acute renal injury by inhibiting macrophage activation [68]. Other experiments have documented that MSC-EVs derived from mouse BM contain immunomodulatory proteins such as programmed death ligand-1 (PD-L1), galectin-1, and transforming growth factor-beta (TGF-). Besides, BM-MSC-EVs have higher levels of cyclooxygenase 2 (COX-2) and prostaglandin E2 (PGE2) contributed to the negatively regulating pro-inflammatory cytokine effects in the splenocytes [69]. Other predominant parts of the exosomal composition, nucleic acids include mRNA, miRNA, transfer RNA, ribosomal RNA, nucleolar RNA, long non-coding RNA (lncRNA), and DNA fragments. Importantly, RNA conveys genetic information, which in turn, affects protein expression and biological activity in the recipient cells [70,72]. Undoubtedly, one of the most important contents of exosomes is miRNAs due to their competence to elicit multifaceted function in human damaged or cancerous tissue by targeting a myriad of molecules and axis. Numerous training has compared the composition of miRNAs between MSC exosomes and their parental MSCs. Correspondingly, the expression of miR-21 and miR-15 involved in the regulation of cardiac functions was meaningfully lower in MSC-EVs in comparison to the parental MSCs [73]. Also, miRNA array analyses have demonstrated that miRNA profiles for human glioma-associated MSC exosomes and human BM-MSC exosomes are altered from their parental cells. Other studies have indicated that the top four miRNAs that are more obviously enriched in MSC exosomes than MSCs include miR-4485, miR-150-5p, miR-6087, and miR-486-5p, and conversely, top four miRNAs, which largely are found in MSCs, involve miR-34a-5p, miR-34c-5p, miR-15a-5p, and miR-136-3p [74]. Other reports signify that exosomal let-7f, miR-20b, and miR-30e-3p levels are modified in the plasma of patients with small-cell lung cancer [75], and also the existence of the close association between ovarian cancer incidence and progress with miR-21 and miR-141 plasma levels has been robustly evidenced [76].

## Exosome isolation methods

Various researches indicate that there are some challenges in the clinical use of exosomes as among various purification processes, there is no standard method for separating exosomes from other micro-particles and also for the separation of various exosome subsets [77, 78]. One of the traditional and most widely used techniques for exosomal isolation is differential centrifugation, which purifies exosomes based on density and size. This method is simple, effective, and economical, but this procedure has a low output and specificity and may also be contaminated with other EVs of a similar dimension. Also, the high speed of centrifugation in this process may damage exosomes. Currently, differential centrifugation has been combined with a sucrose density gradient or sucrose cushions to improve the yield and purity of isolated exosomes [79, 80]. Filtration/ultrafiltration is another option that isolates exosomes based on the pore size of the filter. The filtration method takes less time and troublesome than the differential ultracentrifugation; however, since this approach is based on size, the separation of exosomes from other particles of the same size like apoptotic bodies, or microbubbles is difficult [36, 81]. Size-exclusion chromatography, similar to filtration, isolates exosomes depending on size using columns filled with pore beads. Although the yield of exosome purification is high in this procedure, this technique is time-consuming and is not the epitome for the obtaining of exosomes from large sample volumes [81, 82]. Further, the immunoaffinity strategy has a high purity and yield in exosome purification by binding exosome-associated antigens to antibodies in chromatography columns, magnetic beads, and micro-fluidic systems [83]. On the other hand, though precipitation can also be used for the purification of exosomes, the process is not specific and may co-precipitate other particles and also the chemical treatment may also destroy exosomes thru this process [81]. Moreover, the resuspension of the pellet is complicated and inappropriate for functional use. Overall, although each insolation method has its benefit and downside, its drawback may be dissolved by combining two or more purification procedures and improving both purity and quantity.

## Application of MSC-derived exosomes in regenerative medicine

As known, human MSCs have a great competence to sustain tissue homeostasis as their high potential to home into damaged tissue and their effect on a variety of biochemical and cellular processes such as immune regulation, bioenergy, and tissue regeneration [84,86]. These processes are attributed to direct interactions with different types of cells or by paracrine mechanisms so through the production of soluble biological factors like exosomes [87]. Therefore, a large number of studies have focused on the beneficial protective and therapeutic effect of MSCs in the field of regenerative medicine. Exosomes and other EVs secreted by MSCs have an influential role in sustaining and restoring tissue microenvironment homeostasis supported by the high biochemical potential of their bioactive contents, such as proteins and RNAs [3]. Exosomes as nano-sized particles with less immunoreactivity than whole stem cells possess no potential of differentiating to adult cell lineage or tumor formation, attracting particular attention as a therapeutic vehicle for tissue recovery goal in cell-free based therapies (Table1) [117,119]. Moreover, studies show that exosomes produced by MSCs of different origins contain significantly different functional molecules and exhibit heterogeneous characteristics [120,122]. Exosomes from MSCs from bone marrow, adipose tissue, and umbilical cord, for example, showed a major variation in their proteomic profile and potentially therapeutic. Besides, BM-MSC exosomes have pronounced regeneration capacity by induction of the angiogenesis and also AT-MSC exosomes show the most influential secretory activity and immune response regulation compared with MSCs derived from other sources, while UC-MSC exosomes mostly participate in tissue repair, according to Wang et al. reports [123]. The exact underlying mechanism of MSC-derived exosomes in the restoration of damaged tissue is unclear, while a large number of investigations have suggested that exosomes contribute to the regeneration of damaged tissues by several mechanisms, including (a) the maintenance of endogenous stem cells by proliferating, self-regenerating and differentiating [124, 125]; (b) the induction of regenerative phenotypes by promoting cell proliferation, angiogenesis, and regeneration of nerves [126,129]; (c) the protection of cells from apoptosis by various mechanisms and the amelioration of tissue damage [69]; (d) the attenuation of oxidative stress and the adjustment of the immune response through the delivery of immunomodulatory mediators to damaged tissue [41, 130, 131]. Accordingly, EVs derived from MSCs, especially exosomes, can restore tissue damages and uphold their therapeutic efficacy by transferring biologically active molecules and affecting target molecules, finally regulating the gene expression and phenotype of damaged recipient cells [124, 132,134]. Growing proof shows that MSC-derived exosomes have a diagnostic/therapeutic potential in different pathological conditions, covering cardiovascular diseases, liver, kidney, lung injuries, and neurological disorders [130, 135].

**Table 1:** Therapeutic potential of MSC exosomes in the context of regenerative medicine

*Condition*	*MSC source*	*Isolation method*	*Mechanism*	*Main outcome*	*Ref*
MI	Cardiac MSC	Precipitation at 4C with 5 polyethylene glycol 4000	Transferring miRs	Promoting cardiac angiogenesis and activating cardiomyocyte proliferation (animal study)	[88]
MI	BM-MSC	ExoQuick Exosome Precipitation Solution	Delivering miR-221 and miR-19a	Higher protective effects in cardiomyocytes promoted the growth of cardiomyocytes and prevented cell apoptosis (animal study)	[89, 90]
MI	BM-MSC	Ultracentrifugation (110,000*g*)	Delivering miR-210 that decreases the expression of ephrin-A3	Promoted angiogenesis and cardiac function (animal study)	[91]
Myocardial IRI	HuES9.E1-derived MSC	Chromatography columns	Stimulating anti-apoptotic pathways and also inhibiting pro-apoptotic pathways	Enhanced bioenergetics and pro-survival signaling of cardiac cells (animal study)	[92]
MI	BM-MSC	ExoQuick-TC	n.a	Inducing neovascularization and suppressing inflammatory response (animal study)	[93]
MI	BM-MSC	Ultracentrifugation (200,000*g*)	Delivering MiR-125b-5p	Inhibited apoptosis of cardiomyocyte through inhibition of pro-apoptotic genes p53 and BCL2-antagonist/killer 1 (BAK1) (animal study)	[94]
Autoimmune hepatitis	BM-MSC	Ultracentrifugation (100,000*g*)	Delivering exosomal miR-223	Downregulating various inflammatory genes and proteins and inhibition of hepatocyte death (animal study)	[95]
Hepatic oxidant injury	UC-MSC	Ultracentrifugation (100,000*g*)	Delivering glutathione peroxidase 1 (GPX1)	Suppressed oxidative stress and apoptosis on CCl4-and H_2_O_2_-induced hepatic damage (animal study)	[96]
LF	HuES9.E1-derived MSC	High-performance liquid chromatography (HPLC)	Increasing the expression of cyclin D1 and PCNA as proliferative proteins and enhancing expression of Bcl-xL as an anti-apoptotic factor	Promoting hepatocyte proliferation and inhibiting hepatocyte apoptosis (animal study)	[97]
Liver fibrosis	BM-MSC	Ultracentrifugation (100,000*g*)	Lowering the plasma level of alanine aminotransferase and modulating expression level of inflammatory cytokines and regulatory T cells	Inducing hepatoprotective and anti-inflammatory effects (animal study)	[98]
LF	ESC-MSC	Ultracentrifugation (100,000*g*)	n.a	Enhanced anti-apoptosis, anti-fibrosis, and regenerative capacity (animal study)	[99]
LF	BM-MSC	Ultracentrifugation (100,000*g*)	Delivering Y-RNA-1	Modulated inflammatory response and by inducing multiple anti-apoptotic genes (animal study)	[100]
AD	WJ-MSC	Ultracentrifugation (100,000*g*)	Delivering catalase	Decreasing neuronal oxidative stress and synapse damage (animal study)	[101]
Brain stroke	BM-MSC	Ultracentrifugation (100,000*g*)	Transferring miR-133b to neighboring astrocytes and neuron cells	Increased functional regeneration (animal study)	[102]
SCI	BM-MSC	n.a	Enhanced neuro-vascularization and weaken neuronal apoptosis, as well as prevent inflammation and inhibit the neurotoxic of A1 astrocytes	Boosted SCI functional recovery (animal study)	[103]
SNI	UC-MSCs	Ultracentrifugation	Promoted the generation of axons and Schwann cells, minimized muscular atrophy, and induced anti-inflammatory reactions	Improved functional recovery and peripheral nerve regeneration (animal study)	[104]
SNI	Gingiva MSC	ExoQuick Exosome Precipitation Solution (SBI Systems Biosciences)	Expression of phenotype-related genes, c-Jun, Notch1, GFAP, Sox2 and the stimulation of JNK pathways	Regeneration of peripheral nerve (animal study)	[105]
MS	AT-MSC	Ultracentrifugation (100,000*g*)	Reducing the number of Iba-1-positive microglial cells and also the plasma levels of inflammatory cytokines	Modulated microglial activity and improved immunomodulatory activities (animal study)	[88]
AKI	UC-MSCs	Ultracentrifugation (100,000*g*)	Transferring hepatocyte growth factor (HGF) mRNA	Improved dedifferentiation and proliferation of damaged cells (animal study)	[89]
Kidney IRI	n.a	n.a	Delivering miR-22 for mTOR/HIF feedback interaction pathway and suppressing (PDCD4)/NFKB pathways	Stimulating kidney protection by inducing anti-inflammatory responses and reducing epithelial cell damage (animal study)	[106]
Kidney IRI	WJ-MSC	Ultracentrifugation (100,000*g*)	Reduced kidney cell apoptosis and boosted anti-inflammatory responses	Downregulated CX3CL1 expression and improved kidney function (animal study)	[107]
AKI	UC-MSCs	Ultracentrifugation (100,000*g*)	Activation of the ERK 1/2 pathway and inhibition of the p38MAPK pathway	Suppressing oxidative stress, inducing cell growth, and reducing kidney cell apoptosis (animal study)	[108]
CKD	Urinary MSC	Ultracentrifugation (100,000*g*)	Delivering effective growth factors	Inhibited cell apoptosis and increased vascular regeneration (animal study)	[109]
Kidney artery stenosis	AT-MSC	n.a	Delivering anti-inflammatory cytokine interleukin (IL) 10	Attenuated kidney inflammation and fibrosis, increased kidney blood supply, and suppressed glomerular filtration (animal study)	[110]
Kidney fibrosis	BM-MSC	ExoQuick Exosome Precipitation Solution	Delivering exogenous microRNA let7c	Suppression of kidney inflammation (animal study)	[111]
ALI	UCB-MSC	Ultracentrifugation (100,000*g*)	Delivering VEGF	Restored impaired alveoli function, angiogenic effects, and anti-inflammatory responses and minimized cell apoptosis (animal study)	[112]
ALI	AT-MSC	Ultracentrifugation	Delivering miRNA	Attenuating ALI damage and macrophages polarization (animal study)	[113]
Lung IRI	BM-MSCs	n.a	Transferring miR-21-5p	Inhibited lung edema and improved the function of wound healing (animal study)	[114]
ALI	BM-MSC	Ultracentrifugation	Transferring KGF	Promoted immunomodulatory effects and increased intracellular ATP levels of alveolar epithelial type 2 cell (animal study)	[115]
ALI	BM-MSC	Ultracentrifugation (100,000*g*)	Transferring mitochondrial	Inhibited the production of inflammatory cytokines and enhanced phenotype 2 of alveolar macrophages (animal study)	[116]
PAH	BM-MSC	Ultracentrifugation (100,000*g*)	Alleviated pressure, inhibited right ventricle hypertrophy, and restored pulmonary function	Improved pulmonary artery hypertension (animal study)	[117]

Note: *ALI* acute lung injury, *MI* myocardial infarction, *LF* liver failure, *IRI* ischemia-reperfusion injury, *PAH* pulmonary arterial hypertension, *AKI* acute kidney injury, *CKD* chronic kidney disease, *SCI* spinal cord injury, *SNI* spared nerve injury, *AD* Alzheimers diseases, *MS* multiple sclerosis, *BM* bone marrow, *AT* adipose tissue, *UC* umbilical cord, *UCB* umbilical cord blood, *WJ* Whartons jelly, *miRs* microRNAs, *n.a* not available

### Cardiovascular disease

Cardiovascular disease (CVD) has become a public health concern because it is the main cause of mortality in the world [110, 136]. The ability of endogenous cardiac injury to repair and regenerate is significantly poor due to the insufficient proliferation of existing myocytes concomitant with the weak recruitment and division of resident cardiac progenitor cells [137, 138]. Emerging evidence has shown the cardio-protective role of MSC exosomes in myocardial damage. Ju et al. reported that the delivery of cardiac MSC exosomes after myocardial infarction (MI) enhances cardiac activity by promoting cardiac angiogenesis and activating cardiomyocyte (CMC) proliferation with an unclear mechanism. Cardiac MSC exosomes may enable ischemic myocardium to release chemokines, such as stromal cell-derived factor (SDF) or vascular endothelial growth factor (VEGF), supporting angiogenesis. These exosomes may also upsurge the proliferation of CMC by transferring miRNAs to the ischemic myocardium and by stimulating specific phases of the cell cycle in target cells [88]. Yu et al. have described that exosomes derived from MSC transduced by the growing family of related transcription factor (GATA)-4 genes have enriched various miRNAs, such as miR-221 and miR-19a, which are capable of generating higher protective effects on CMC and also the regeneration of ischemic injury. The miR-221 could improve the survival of myocytes in ischemic injury by reducing the expression of the P53-upregulated apoptosis modulator (PUMA), a member of the B cell lymphoma 2 (Bcl-2) proapoptotic protein families. Also, miR-19a could attenuate the expression of phosphatase and tensin homolog (PTEN), a mediator of Akt and extracellular signal-regulated kinase (ERK) survival signaling pathway, thereby leading to the growth of CMC and preventing cell apoptosis in ischemic myocardium by activating these signaling pathways [89, 90]. Additionally, MSC exosomes contain a high amount of miR-210 that could diminish the expression of ephrin-A3, an angiogenesis suppressor molecule, in endothelial cells to promote angiogenesis and cardiac function in mouse MI models [91]. Another study suggested that MSC exosomes had a direct effect on cardiac cells and enhanced their bioenergetics and survival by reducing oxidative stress, enhancing the production of ATP and NADH, and stimulating anti-apoptotic pathways through Akt and glycogen synthase kinase-3 (GSK3) phosphorylation, and also mitigation of the activation of the proapoptotic pathways mediated by downregulation of c-Jun NH2-terminal kinase (c-JNK). MSC exosomes also could restore cardiac activity and reduce infarct size in the ischemic/reperfuse mouse model of the myocardium [92]. Furthermore, Teng et al. demonstrated that MSC exosomes could recover heart function and repair post-myocardial infarction via inducing neovascularization and suppressing inflammatory response after ischemic injury [93]. In another study, Zhu et al. reported that exposure of BM-MSC to the hypoxia culture medium altered their exosomal content compared to the normoxia culture medium. The miRNA content was shown to be significantly different between normoxia-exposed BM-MSC exosomes (nor-exosomes) and hypoxia-exposed BM-MSC exosomes (hypo-exosomes). Analysis implied that anti-apoptotic miR-125b-5p was one of the most enriched miRNAs in hypo-exosomes with high therapeutic effectiveness in the treatment of MI. Correspondingly, systemic administration of hypo-exosomes inhibited apoptosis of CMC through inhibition of proapoptotic genes p53 and bcl2-antagonist/killer 1 (BAK1) and consequently improved ischemic heart repair in the rodent MI model [94]. Besides, reports have indicated that cardiac MSC-EVs facilitate strong proangiogenic behaviors as well as enhanced cell proliferation, tube forming, and survival of human umbilical vein endothelial cells (HUVECs). These proangiogenic effects of human cardiac MSC-EVs were mainly mediated by positive regulation of the angiopoietin-1 (Ang1) / tyrosine kinase Tie-2 signaling pathway [139]. Other studies have shown that exposure to PDGF enhanced the proangiogenic activity of EVs secreted by AT-MSCs. In EVs derived from PDGF-treated MSCs, the levels of proangiogenic c-kit and stem cell factor (SCF) improved and consequently resulted in induced angiogenesis in vitro and in vivo [140].

### Liver injury and fibrosis

Several studies have also revealed that MSC exosomes possess the ability to rescue liver injury and fibrosis through the delivery of various bioactive molecules to hepatocyte cells and other cells in the liver tissue, and thereby change their function and/or phenotype. MSC exosomes have substantial hepatoprotective effects related to exosomal miRNAs, especially miR-223. MiR-223 plays a vital role in the modulation of the immune system as well as the protection of the liver by downregulating various inflammatory genes and proteins, such as cytokines and nucleotide-binding domain and leucine-rich repeat pyrin containing 3 (NLRP3) and also caspase-1. Exosomal miR-223 suppresses NLRP3/caspase-1 signaling pathway and pyroptosis resulting from this signaling pathway, which may minimize liver inflammation and hepatocyte death in the autoimmune hepatitis animal model [95]. Similarly, Yan et al. have already found that glutathione peroxidase 1 (GPX1) containing MSC exosomes could suppress oxidative stress and apoptosis in carbon tetrachloride (CCl4)-and H2O2-induced hepatic damage and finally restore liver function in vivo. The GPX1 is a main antioxidant enzyme that reduces the formation of reactive oxygen species (ROS) through different mechanisms in inflamed liver tissue. Too, MSC-derived exosomes could increase the spread of hepatocytes and decrease their apoptosis through various pathways [96]. Tan et al. reported that MSC exosomes improved hepatic regeneration of CCl4-induced liver damage in a mouse model through promoting hepatocyte proliferation and inhibiting their apoptosis. They found that MSC exosomes stimulate the hepatocyte cell cycle by upregulation of cyclin D1 and proliferating cell nuclear antigen (PCNA) concurrently suppression of the apoptosis of liver cells by increasing the expression of anti-apoptotic Bcl-xL [97]. In parallel, Tamura et al. investigated the inhibitory effect of MSC-derived exosomes on immune-induced liver damage via injection of concanavalin A (con A). They found that MSC-derived exosomes had hepatoprotective and anti-inflammatory competencies through reducing the expression level of proinflammatory cytokines along with promoting the expression of anti-inflammatory cytokines and the frequency of regulatory T cells [98]. To prolong the bioavailability of MSC-derived exosomes in chronic liver disease, Mardpour et al. encapsulated exosomes in polyethylene glycol (PEG) hydrogels to sustain their release in the systemic circulation. They found that hydrogel-mediated delivery caused boosted accumulation of exosomes in fibrotic tissue over prolonged periods, enabling superior anti-apoptosis, anti-fibrosis, and regenerative capacity over free-exosome delivery [99]. Besides, evaluation of the potential of MSC exosomes as a therapeutic approach for tissue restoration and functional recovery by modifying inflammatory response and inducing multiple anti-apoptotic genes in the experimental acute hepatic failure model suggested that MSC-derived exosome intravenous injection resulted in upregulated Y-RNA-1 as a non-coding RNA in damaged tissue, which in turn, supported defensive mechanisms in the microenvironment of the injured liver [100].

### Neurological diseases

Concerning the observations, restoring deteriorated neural tissue remains a big problem in regenerative medicine due to a complication of the physiology system and a limited ability to self-regenerate [101, 141]. A wide spectrum of reports has demonstrated that MSC exosomes have promising therapeutic potential in neurological disorders, such as neurodegenerative diseases. Recent researches have shown that MSC exosomes can protect hippocampal neurons by decreasing neuronal oxidative stress and synapse damage in an animal model of Alzheimers disease (AD). Exosomes contain multiple active enzymes, in particular catalases, which can reduce the formation of ROS in hippocampal neurons, and thereby protect them from AD and other neurodegenerative conditions [142]. As well, Xin et al. proposed that MSC exosomes induced the remodeling of neuritis and subsequently facilitated functional regeneration in the rat stroke model via miR-133b transfer to adjacent astrocytes and neuron cells [102]. In another study, intravenous administration of MSC exosomes improved neuro-functional recovery and boosted post-stroke neurogenesis and neurovascular remodeling in the rat model. Liu et al. indicated that BM-MSC exosomes could enhance neurovascular development and attenuate neuronal apoptosis. Moreover, these exosomes inhibited neuroinflammation and also suppressed the neurotoxic and reactive state of A1 astrocytes following traumatic spinal cord injury (SCI) in rats, implying their competence to exploit for SCI therapy [103]. Additionally, exosomes derived from MSCs could provide a significant protective role in peripheral nerve damage. In this regard, exosomes derived from UC-MSCs could aggregate in nerve defect, promote the generation of axons and Schwann cells, minimize muscular atrophy, and exert anti-inflammatory reactions in injured nerve tissues by downregulating proinflammatory cytokines (IL-6 and IL-1) concomitant with upregulating anti-inflammatory cytokines (IL-10). Therefore, UC-MSC exosomes could establish a favorable stromal condition for increased functional recovery and amelioration of sciatic nerve defects in rats [104]. Mechanistically, Mao et al. suggested that gingiva MSC exosomes promote regeneration of peripheral nerve via upregulation of genes participated in Schwann cell dedifferentiation or repair, including c-Jun, notch1, glial fibrillary acidic protein (GFAP), and SRY (sex-determining region Y)-box 2 (Sox2) and the stimulation of c-Jun N-terminal kinase (JNK) pathways in Schwann cells. Hence, these findings provide proof of the concept that gingiva MSC exosomes activate repair phenotype in Schwann cells and promote their proliferation and migration [105]. Besides, AT-MSC exosomes demonstrated inhibitory effects on inflammation-mediated demyelinating disease, in particular, multiple sclerosis (MS). Exosomes also greatly modulate microglial activity by altering microglial cell morphology and reducing the number of Iba-1-positive microglial cells in the brains of the Theilers murine encephalomyelitis virus (TMEV)-infected mice. Regarding observations, administration of exosomes intravenously improved immunomodulatory activities by reducing the plasma levels of inflammatory cytokines, substantially the concentration of Th1 cell-generated IFN- and Th17 cell-produced IL-17A, resulted in ameliorated motor deficits of TMEV-infected mice [88]. As well, systemic administration of prostaglandin E2 receptor 4 (EP4) antagonists induced MSC-EV secretion and increased the production of anti-inflammatory cytokines and other proteins such as IL-2, IL-10, RANTES, vascular endothelial growth factor (VEGF), and brain-derived neurotrophic factor (BDNF) which could alleviate memory and learning deficiencies, improve blood-brain barrier integrity and anti-inflammatory responses, and also elicit functional recovery in a mouse model with hippocampus damage [143].

### Kidney disease

Preclinical researches have demonstrated that MSC exosomes have a marked capacity to rescue acute and chronic kidney disease via the secretion of various mRNAs, miRNAs, and immunosuppressive factors into the tissue environment [127, 144, 145]. The molecular mechanisms responsible for kidney regeneration rely on the control of immune responses, the prevention of apoptosis and necrosis of epithelial tubular cells, and the reduction of oxidative stress in the kidney tissue. Ju et al. have shown that MSC exosomes improved dedifferentiation and proliferation of damaged tubular cells by transfer of hepatocyte growth factor (HGF) mRNA and enhanced HGF synthesis, thereby accelerating kidney regeneration and delaying fibrosis [89]. Also, another study verified the role of MSC-generated miRNAs in kidney protection by suggesting the anti-inflammatory ability of miR-22 in the relief of I/R-induced acute kidney injury (AKI). In detail, miR-22 suppresses the programmed cell death 4 (PDCD4)/ nuclear factor-B (NF-B) pathways required for dendritic cell (DC) maturation, which consequently attenuates its potential for the secretion of proinflammatory cytokines. MiR-22 also reduces epithelial cell damage via targeting the mammalian target of rapamycin (mTOR)/ hypoxia-inducible factor (HIF) feedback interaction pathway, enabling AKI recovery [106]. Further, Zou et al. found that the exosomes derived from human Whartons Jelly MSC (WJ-MSC) downregulated chemokine C-X3-C motif ligand 1 (CX3CL1) expression and improved kidney function in rats with kidney ischemia-reperfusion injury (IRI). They found that the exosomes could reduce kidney cell apoptosis and raise the proliferation of these cells concomitant with the enhancement of anti-inflammatory responses by decreasing the macrophage frequencies in wounded sites [107]. Moreover, human UC-MSC exosomes could potently improve cisplatin-induced AKI in laboratory models by suppressing oxidative stress upon decreasing oxidant enzymes and increasing antioxidant enzymes cellular levels and also through mitigation of kidney cell apoptosis by inhibition of the p38 mitogen-activated protein kinase (MAPK) pathway [108]. Besides, intra-kidney delivery of MSC exosomes could restore kidney injury in pigs with metabolic syndrome and kidney artery stenoses possibly mediated by increased kidney blood supply, suppressed glomerular filtration, modified kidney inflammation, and abrogated kidney fibrosis and medullary oxygenation, supporting kidney normal function. It has been supposed that MSC exosomes elicited these protective effects upon IL-10 upregulation as an anti-inflammatory cytokine [110]. Another research indicated that BM-MSC exosomes could deliver exogenous microRNA let7c with strong anti-fibrotic activity via suppression of kidney inflammation in rodent models [111]. Respecting to the glial cell line-derived neurotrophic factor (GDNF) neurotrophic property, exosomes derived from GDNF-overexpressed AT-MSCs were found to stimulate angiogenesis and enhance peritubular capillary loss in tubulointerstitial fibrosis in vivo by activating the SIRT1 signaling pathway and inducing the production of phosphorylated endothelial nitric oxide synthase (p-eNOS) [146]. Moreover, in an experimental model of type I diabetes, intravenous administration of MSC exosomes significantly ameliorated diabetes-induced kidney injury by suppressing apoptosis of podocytes and tubular epithelial cells and inducing tubular vascular survival and regeneration. MSC exosomes contained angiogenesis-related factors such as transforming growth factor- (TGF-), angiogenin, and bone morphogenetic protein-7 (BMP-7) played central roles in these processes [147].

### Lung diseases

**Fig. 3:**
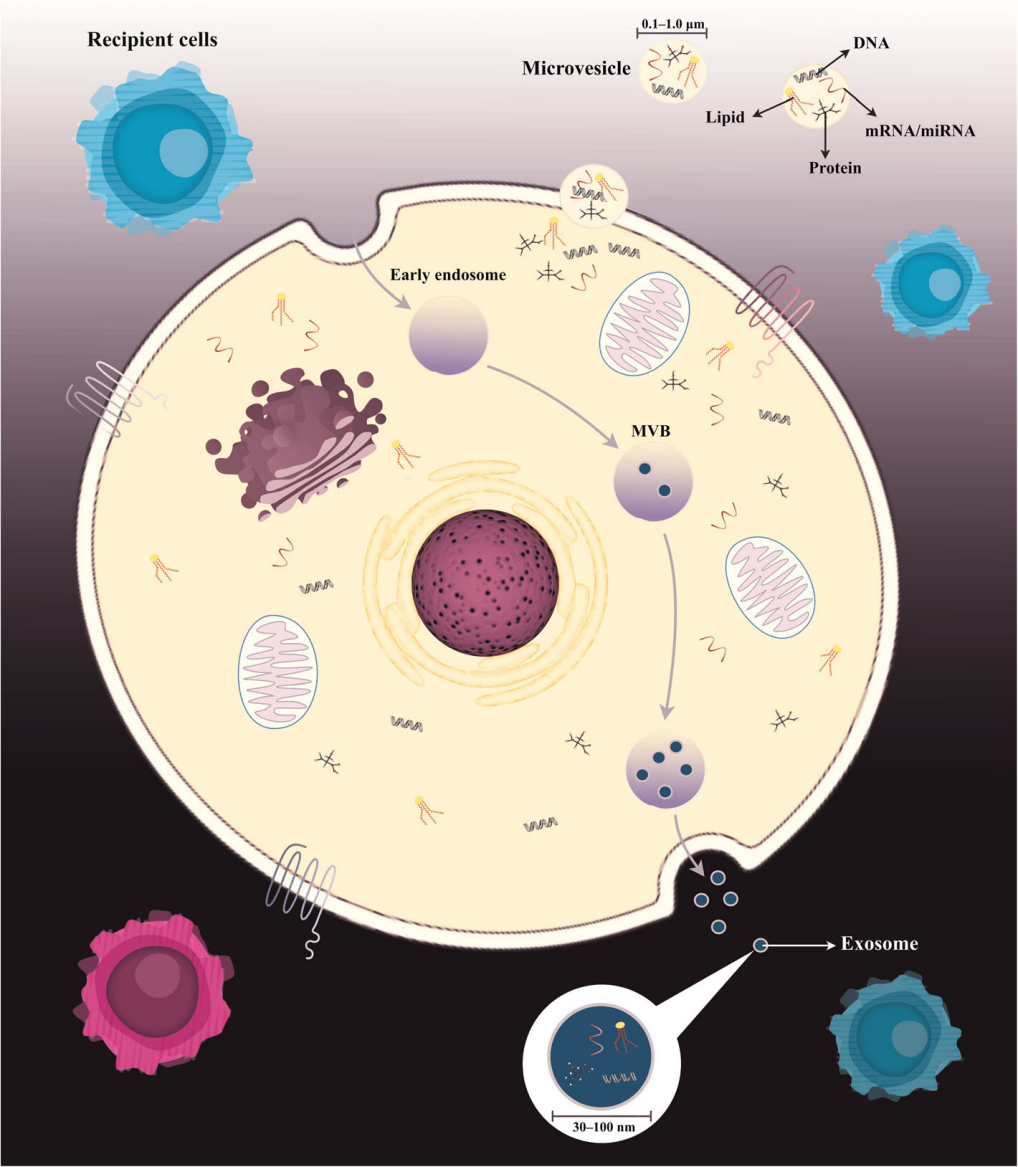
Extracellular vesicle (EVs) biogenesis. Exosomes are smaller luminal vesicles (30100nm in diameter) crated from late endosomes that are shaped by inward budding of the limited multivesicular body (MVB) membrane; however, microvesicles (MVs) are small membrane vesicles (0.11m in diameter) secreted from the cell membrane surface. The content, or cargo, of EVs, includes lipids, nucleic acids, and proteins, in particular, proteins related to the plasma membrane, cytosol, and those that participated in lipid metabolism

Rising preclinical evidence shows that MSC-derived exosomes have substantial therapeutic potential in the regeneration and rehabilitation of multiple lung diseases by mediating different molecular pathways and affecting different target cells in the lung tissue environment, such as lung endothelial and epithelial cells and immune cells, leading to the desired therapeutic outcomes following injection by various routes in particular intravenous or inhalation route [148, 149] (Fig.3). Ahn et al. recently found that VEGF plays a crucial function in the protection of exosomes in hyperoxic lung injuries. Correspondingly, exosomes generated by human UC-MSCs have significant protective efficacy by restoring impaired alveoli function and angiogenic effects, lessening cell apoptosis, and suppressing macrophages and proinflammatory reactions in newborn hyperoxic lung injuries in a rat model [112]. Compartmental analysis of the therapeutic capacity of fresh MSC exosomes and aged MSC exosome demonstrated that fresh exosomes had superiority over aged exosomes in terms of the attenuating acute lung injury (ALI) and macrophage polarization because of the existing differences in miRNA content [113]. Besides, Li et al. reported that intratracheal administration of MSC-generated exosomes had a protective effect in the treatment of the murine model with lung IRI by transporting miR-21-5p, as evidenced by inhibited lung edema and improved tissue regeneration by downregulating PTEN and PDCD4. MSC exosomes also shifted alveolar macrophage polarization from M1 to M2 phenotype caused inhibited proinflammatory responses in a miR-21-5p-dependent manner in the murine ischemia-reperfusion injury (IRI) model lung [114]. Interestingly, Monsel and his colleagues found that MSC exosomes could improve ALI induced by *E. coli* pneumonia by secretion of the keratinocyte growth factor (KGF) and binding to its CD44 receptor on the surface of monocytes and alveolar epithelial cells in the murine model. Administration of MSC exosomes intravenously improved immunomodulatory effects in bacterial pneumonia injuries by improved phagocytosis potential of monocytes and decreased inflammatory cytokine secretion. In particular, exosomes restored alveolar epithelial type 2 cell metabolism by increasing their intracellular ATP levels [115]. In another study, MSC exosomes mediated mitochondrial transfers, which in turn, inhibited the production of inflammatory cytokines and enhance phenotype 2 of alveolar macrophages to mitigate ALI in vivo [116]. Additionally, MSC exosomes could improve pulmonary artery hypertension (PAH) through alleviating mean pulmonary artery pressure (mPAP) and mean right ventricle pressure (mRVP), inhibiting right ventricle hypertrophy, and restoring pulmonary functions in the murine PAH models [117]. Besides, hepatocyte growth factor (HGF) derived from MSC-MVs has been shown to reduce pulmonary microvascular endothelial paracellular and transcellular permeability by facilitating the expression of junction proteins VE-cadherin and occluding under pathological conditions such as acute lung injury [150]. Moreover, MSC-MVs prevented endothelial cell apoptosis, increased IL-10 expression, and reduced IL-6 expression in endothelial cell culture media via an HGF-dependent action in vitro. In another study, the therapeutic capacity of hUC-MSC-EVs versus hUC-MSCs on the treatment of chronic obstructive pulmonary disease (COPD) in a rat model was investigated. Both hUC-MSC-EVs and hUC-MSCs ameliorated peribronchial and perivascular inflammation and thus decreased alveolar septal thickening in the emphysematous lung of COPD by decreasing the synthesis of protein kinase C zeta and NF-B subunits p50 and p65, which control several pathways associated with innate and adaptive immune response [151].

As well, another study in 30 participants with COPD indicated that administration of MSC exosomes was well tolerated because none of them experienced any unwanted events [152]. Moreover, investigation of the safety and efficacy of exosomes (ExoFlo) derived from allogeneic BM-MSCs in 24 patients with severe 2019 coronavirus disease (COVID-19) revealed that systemic injection of the ExoFlo caused restored patients clinical condition and oxygenation without any serious events during 2weeks follow-up, suggesting MSC exoxomes as a capable therapeutic candidate for severe COVID-19 [153].

## Double-edged sword role of MSC-derived exosomes in tumors

**Fig. 4:**
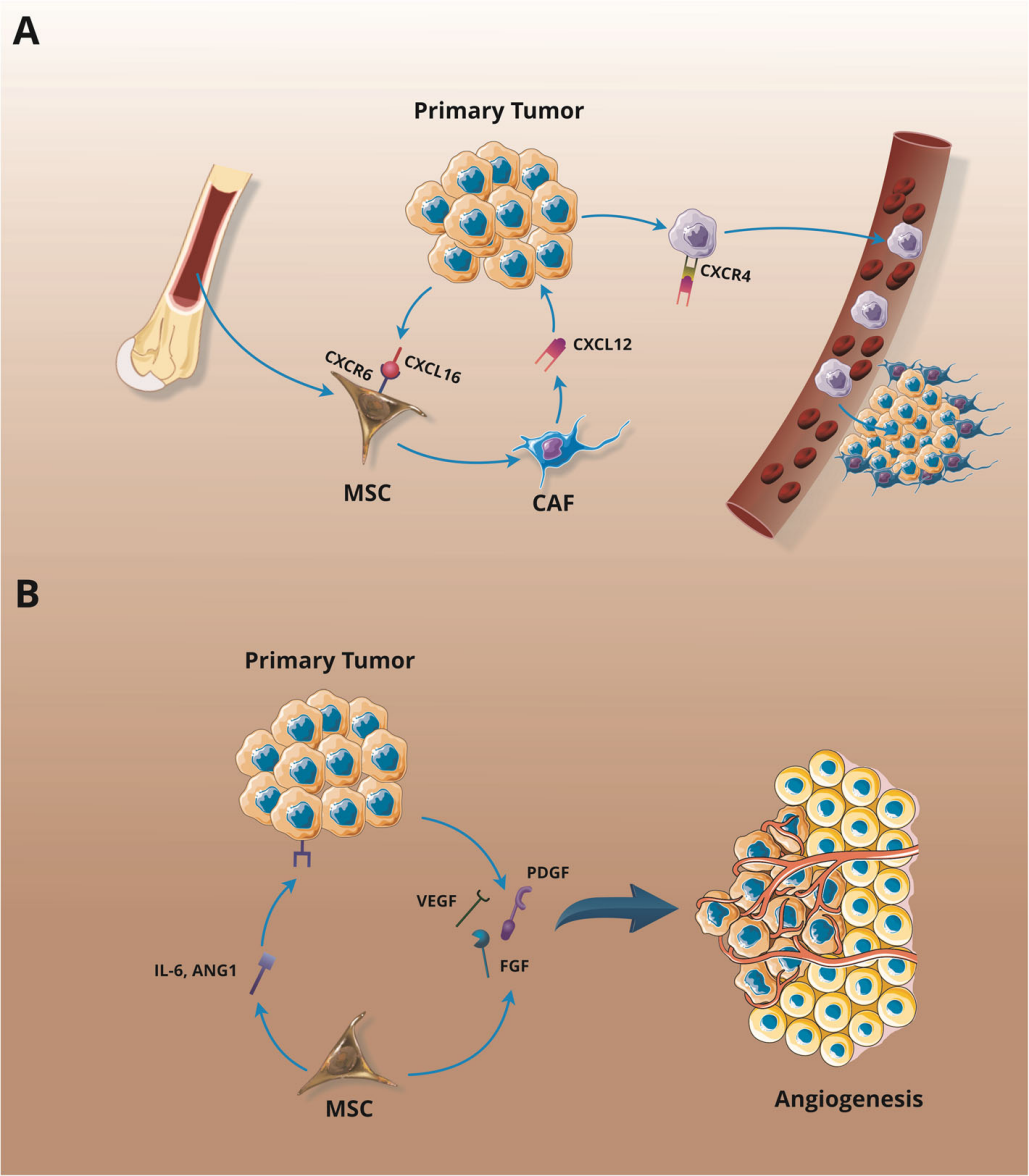
MSCs role in tumor metastasis (**a**) and angiogenesis (**b**). **a** The cancer cells release CXCL16 which can elicit the recruitment of MSCs in the tumor site. Upon the bindings of CXCL16 to CXCR6 on MSCs, they are transformed into CAFs producing high levels of CXCL12. CXCL12 stimulates the EMT process on cancer cells that promote CXCR4 expression in cancer cells, leading to their metastasis. **b** The MSCs commonly generate and release varied angiogenic mediators surrounding angiopoietin-1 (ANG-1) and IL-6 that, in turn, provoke the release of VEGF and other angiogenic factors stimulating tumor angiogenesis. CAF; cancer-associated fibroblast, EMT; epithelial-to-mesenchymal transition, MSC; mesenchymal stem/stromal cell, FGF; fibroblast growth factor, ANG-1; angiopoietin-1, PDGF; platelet-derived growth factor, VEGF; vascular endothelial growth factor

The microenvironment around the tumor cells includes extracellular matrix (ECM) and soluble factors along with various tumors stromal cell types, such as MSCs, fibroblasts, immune cells, epithelial cells, and endothelial cells, which have a potent effect on tumor growth and progression modulation [154,156]. The MSCs are a significant constituent of tumor stromal cells which may be recruited at tumor sites with ambiguously defined biological mechanisms and may support the formation of a tumor-associated microenvironment. The crosstalk of MSCs with tumor cells within the tumor stroma affects various cancer features [156] and can exert contradictory functions in various tumors. These investigations have shown that MSCs can either support tumor development and angiogenesis (Fig.4), but can also suppress tumor growth and progression [157, 158]. Recent studies have suggested that MSC-derived exosomes have a potential role in the shaping of the communication between MSCs and tumor cells and also evidenced the regulatory effects of MSCs on the microenvironment of the tumor by distributing soluble factors and genetic biomolecules (Table2) [124, 178].

**Table 2:** Dual role of MSC exosomes in the context of cancer therapy

*Donor cells*	*Cell line/condition*	*Exosome content*	*Function*	*Pathway*	*Ref*
Promoting effect					
BM-MSCs	MM	miR-15a	Enhanced MM cell proliferation (animal study)	n.a	[159]
MSCs	BRCA cells	miR-222/223	Supported the survival and growth of cancer cells (in vitro study)	Promoted quiescence in a subset of cancer cells and confers drug resistance	[160]
AT-MSCs	HUVECs	miR-125a	Promoted angiogenesis in vitro and in vivo	Repressed the expression of the angiogenic inhibitor delta-like 4 (DLL4)	[111]
AT-MSCs	Breast cancer MCF7 cells	n.a	Promoted MCF7 migration (in vitro study)	Activating Wnt signaling pathway	[161]
BM-MSCs	Osteosarcoma MG63 and gastric SGC7901 cells	n.a	Supported MG63 and SGC7901 cell growth (in vitro study)	Activating Hedgehog signaling pathway	[162]
AT-MSCs	HMEC	c-KIT and SCF	Induced angiogenesis (animal study)	Promoted survival, migration, and angiogenesis	[140]
MSCs	Gastric cancer	n.a	Stimulated the proliferation and migration of gastric cancer cells ex vivo (animal study)	Activation of the Akt pathway	[163]
AT-MSCs	Glioblastoma U87MG cells line	n.a	Induced cell proliferation (in vitro study)	n.a	[164]
GC-MSCs	Gastric cancer cells	miR-214, miR-221, and miR-222	Promoted tumor proliferation and migration (in vitro study)	Downregulating several tumor suppressor target genes	[165]
Inhibitory effect					
MSCs	Breast cancer cells	miR-379	Inhibition of angiogenesis, tumor amplification, and metastasis (in vitro study)	Regulation of COX-2 mRNA and protein	[166]
UC-MSCs BM-MSCs	Glioblastoma U87MG cells	n.a	Decreased cell proliferation and induced apoptosis of glioblastoma cells (in vitro study)	n.a	[164]
MSCs	Gastric cancer	n.a	Induced drug resistance in gastric cancer cells (animal study)	Triggered the activation of calcium/calmodulin-dependent protein kinases (CaM-Ks) and Raf/MEK/ERK kinase cascade	[167]
BM-MSCs	Glioma	miR-146b	Reduced glioma cell invasion, migration, viability, and expression of EGFR (animal study)	Decreased EGFR and inhibited NF-B, as well as its upstream regulator TRAF6	[168]
MSCs	Breast cancer	miR-16	Suppressed angiogenesis (animal study)	Downregulated VEGF expression in tumor cells in vitro and in vivo	[169]
WJ-MSCs	Burkitts lymphoma cells	miR-34a and let-7 family	Arrested cell division of lymphoma cells in the S phase and induced apoptosis by using oxidative stress pathways (in vitro study)	Alteration of antioxidant enzymes	[170]
AT-MSCs	Hepatocellular carcinoma (HCC)	miR-122	Increased the sensitivity of HCC cells to chemotherapeutic agents and induction of apoptosis and cell cycle arrest (animal study)	Reduced expression of cyclin G1 (CCNG1 ) and inhibition of the PI3K/Akt pathway	[171]
BM-MSCs	Breast cancer	miR-23b	Induced dormant phenotypes (animal study)	Suppression of MARCKS gene	[172]
MSCs	Breast cancer cells	miR-100	Suppressed angiogenesis (in vitro study)	Modulated the mTOR/HIF-1/VEGF signaling axis	[173]
WJ-MSCs	Bladder cancer cells	n.a	Inhibited proliferative viability and induced apoptosis (in vitro study)	Downregulated phosphorylation of Akt and upregulated cleaved Caspase 3	[174]
BM-MSCs	Oral cancer	miR-101-3p	Inhibited proliferation, invasion, migration, and tumor growth (animal study)	Downregulated type X collagen gene	[175]
BM-MSCs	Leukemia THP-1 cells	miR-222-3p	Inhibited cell proliferation and promoted cell apoptosis (in vitro study)	Negatively regulating IRF2-INPP4B signaling	[176]
AT-MSCs	Ovarian A2780 and SKOV-3 cancer cells	miRNAs	Inhibited the proliferation and growth (in vitro study)	Upregulated pro-apoptotic proteins, as well as downregulated the anti-apoptotic protein	[177]

Note: *BM* bone marrow, *AT* adipose tissue, *UC* umbilical cord, *WJ* Whartons jelly, *miRs* microRNAs, *GC-MSCs* gastric cancer-derived MSCs, *MM* multiple myeloma, *HMEC* human microvascular endothelial cells, *HUVECs* human umbilical vein endothelial cells, *MARCKS* myristoylated alanine-rich C-kinase substrate, *IRF2-INPP4B* interferon-regulatory factor 2 -inositol polyphosphate-4-phosphatase type-II, *n.a* not available

### Tumor growth

Tumor progression and angiogenesis are related to a range of growth factor receptors and the activation of signaling pathways associated with these receptors. Stimulation or phosphorylation of the intracellular domain of these receptors leads to downstream signaling via Akt, PKC, and ERK kinase pathways which promote malignant cell proliferation and migratory phenotypes in tumor cells [179]. MSC exosomes are recognized to deliver their content to adjacent tumor cells and to affect tumor development with tumor-supporting or tumor-inhibiting mechanisms.

#### Tumor-supporting effects of MSC exosomes

Extensive research has related the transfer of tumor-associated factors present in MSC-derived exosomes to cancer cell proliferation promotion [37]. Vallabhaneni et al. indicated that exosomes secreted from stressed hMSCs stimulated the proliferation of breast cancer cells and metastases via the transmission of significant quantities of tumor-supportive miRNAs, such as miR-21 and miR-34a, and various proteins that are recognized as tumor-promoting factors [180]. Also, Dong et al. found that miR-410 from hUC-MSC-derived exosomes directly suppressed PTEN expression in lung adenocarcinoma cells and supported the growth of lung adenocarcinoma through enhanced proliferation and reduced apoptosis of these cells [181]. Genetic and protein composition has also been found to differ between multiple BM-MSC-derived exosomes and normal BM-MSC-derived exosomes. Lower levels of the tumor suppressor miR-15a and higher levels of adhesion molecules, oncogenic proteins, and cytokines in multiple myeloma BM-MSC-derived exosomes could promote the progression of multiple myeloma disorders, showing that the various origins of MSC-derived exosomes result in unexpected therapeutic outcome [159]. Moreover, Zhu et al. have demonstrated that MSC-derived exosomes increased the expression of VEGF in tumor cells through the activation of ERK1/2 and p38 MAPK pathways resulted in increased tumor growth and angiogenesis in vivo [182]. Other investigations revealed that human BM-MSC exosomes stimulated the Hedgehog (Hh) signaling pathway by influencing the expression of various components of this signaling cascade in osteosarcoma MG63 and gastric cancer SGC7901 cells and promoting tumor progression [162].

#### Tumor-inhibiting effects of MSC exosomes

Oppositely, the inhibitory effects of exosomes on tumor growth have also been reported. MiR-146b in MSC exosomes can suppress tumor growth in culture. MiR-146b from MSC exosomes binds to epidermal growth factor receptor (EGFR) mRNA, and eventually reduced growth, migration, and invasion of cancer cells in culture [168]. Intra-tumoral injection of MSC-derived exosomes containing miR-146b could also suppress glioma development in a rat primary brain tumor model [168]. Similarly, Takahara et al. discovered that AT-MSC exosomes greatly suppressed human prostate cancer cell proliferation by delivering miR-145 to these cells. Moreover, miR-145 as a tumor suppressor could inhibit the progression of prostate cancer and induce apoptosis in these cells via activating the caspase-3/7 mediated apoptosis pathway and suppressing the anti-apoptotic activity of Bcl-xL [183]. Likely, human WJ-MSC secretome showed anti-proliferative and apoptosis-inducing effects on human bladder cancer T24 cells in vitro and bladder cancer experimental models mediated by restraining phosphorylation of Akt protein kinase and upregulating p53/p21 and cleaved caspase 3 [174]. It seems that the differences in the periods of MSC expansion, the culture medium compounds, and the MSC passage number may act as influential factors that determine the consequence of the employment of exosomes for tumor therapy. Consistent outcomes in the performance and content of exosomes can therefore be obtained by monitoring the preparation conditions of MSCs [164, 179].

### Tumor angiogenesis and metastasis

Neovascularization is known to be a critical biochemical step in tumor development and metastases. Both tumor and stromal cells in the tumor microenvironment participate a pivotal role in the regulation of tumor angiogenesis through the secretion of diverse signaling molecules and factors [184, 185]. Studies in different tumors have shown that exosomes produced from different cell types, in particular MSCs, could affect angiogenic response by targeting diverse molecules of angiogenetic signaling pathways. Latest experiments have shown that MSC-derived exosomes release components that act as angiogenic stimuli or angiogenic repressors. Thus, conflicting effects of MSC-derived exosomes on angiogenesis in different tumors may arise due to several elements, for example, the variance in the form of MSCs (i.e., BM-MSCs versus tissue-derived MSCs), the variability of MSC growth conditions, and the different dosage or interval of MSC infusion. Exosomes derived from MSCs have been shown to dramatically increase the expression of VEGF in tumor cells by activation of ERK1/2 and p38 MAPK pathways, sustaining tumor growth, and angiogenesis [182]. Also, AT-MSC-derived exosomes can deliver miR-125a as a proangiogenic factor to endothelial cells and consequently facilitate the development of endothelial tip cells and angiogenesis by suppressing the expression of repressing angiogenic inhibitor delta-like 4 (DLL4) expression [111]. Conversely, exosomal miR-16 has been reported to significantly reduce VEGF expression in breast cancer cells leading to the inhibition of angiogenesis in vitro and in vivo [169]. Additionally, Pakravan et al. revealed that the transfer of miR-100 from human BM-MSC-derived exosomes to breast cancer cells decreased VEGF expression and secretion by modulating the mTOR/HIF-1/VEGF signaling axis [173]. MSC-derived exosomes can also be used as an efficient modulating angiogenetic factor for tumor therapy.

Tumor metastases initiate with the separation of the tumor cells from the primary neoplasm and moving to the distant organ site, which includes a series of distinct multistep biological events. Any of these events are influenced by the biological and molecular alteration of cancer cells and the collaboration of stromal cells in the microenvironment of the tumor [186, 187]. Emerging results have confirmed that MSC-derived exosomes contribute to tumor metastases and the development of a pre-metastatic niche [188]. Studies in MCF-7 breast cancer cells revealed that exosomes secreted by AT-MSCs could successfully upregulate Wnt/-catenin signaling pathways and increase expressions of Wnt target genes such as Axin2 and Dickkopf-1 (Dkk1), thereby facilitating the progression and migration of cancer cells [161]. Similarly, MSC-derived exosomes promote the migration and invasion of HGC-27gastric cancer cells via enhancing the development of epithelial-mesenchymal transition (EMT) and boosting the expression of mesenchymal markers, and reducing the expression of epithelial markers in HGC-27 cells. It was further demonstrated that the expression of OCT4 and Lin28B increased in HGC-27 cells after treatment with MSC exosomes. Besides, these exosomes promoted the development and metastatic potential of HGC-27 cells by activating the Akt signaling pathway [163].

On the other hand, several reports are demonstrating that treatment with MSC-derived exosomes could prevent tumor migration and invasion. Shimbo et al. found that the transfer of miR-143 enriched exosomes into osteosarcoma cells may substantially alter the physiological features of osteosarcoma cells and suppress the migration ability of these cells [189]. Moreover, Ono et al. have demonstrated that the exosomal transfer of miR-23b from the BM-MSCs to breast cancer cells suppressed the target gene, myristoylated alanine-rich C-kinase substrate (MARCKS), which is responsible for promoting cell cycling and motility. Therefore, these findings suggest that exosomes maintained breast cancer cells in dormancy and prevents their recurrence [172]. In conclusion, these observations show that MSC-derived exosomes affect cell-to-cell communication from different pathways which may be essential for the modulation of tumor metastases.

### Tumor cell-free therapy by MSC exosomes

Exosomes are recognized as promising natural nanovesicles for clinical therapeutic and diagnostic applications due to their effective biocompatibility and biodistribution properties. Further, engineered/modified exosomes can be used as effective therapeutic options for cancer because of their properties as natural important intercellular contact mediators and their innate ability in packaging and transition of therapeutic agents such as medicines, certain unique mRNAs, miRNAs, regulatory miRNAs, lipids, and proteins [190]. Over the past decades, the use of MSCs and their unlimited capacity in the development of exosomes as a nanocarrier in anticancer therapy have been extensively studied. It has been shown that the induction of MSCs to secrete exosomes loaded with antagomyR-222/223 and systemic administration of these exosomes could sensitize breast cancer cells to carboplatin-based therapy and enhance the cell dormancy of cancer cells to increase host survival in breast cancer [160]. Furthermore, it has been reported that MSC-derived exosomes loaded with anticancer protein, tumor necrosis factor (TNF)-related apoptosis-inducing ligand (TRAIL), elicited considerable apoptosis in a series of cell lines [191]. Besides, the use of exosomes as a natural drug delivery vehicle has several benefits over artificial delivery systems as exosomes are derived from natural cell supplies with low immunogenicity and reduced toxicity following delivery to the recipient cells. On the other hand, exosomes have a phospholipid bilayer structure that expresses ligands and receptors that can bind with specific ligands and receptors or target cell membranes and internalize their encapsulated contents to target cells. The small size of exosomes increases their ability for long circulation and thus inhibits deterioration or elimination of reticuloendothelial (RES) organs, enhances their extravasation from the blood vessels, and ultimately penetrates deep tissue. The capacity of exosomes in the sustained release of a specific amount of drugs increases their therapeutic potency due to extended circulation of drugs and their accumulation in the recipient cells [192, 193]. Meanwhile, MSC-released exosomes loaded with paclitaxel (PTX) demonstrate the potent anticancer activity by inhibiting the proliferative activity of the CFPAC-1 pancreatic cell line [194]. Also, it has been shown that PTX-loaded MSC-derived exosomes hindered the development of different tumor cell populations in vitro and obstructed the appearance of metastasis in primary tumors in vivo [195]. Despite the promising future of exosomes as a drug vehicle for the treatment of different forms of the tumor, the lack of consistent protocols for the production, isolation, and purification of exosomes leads to contradictory findings that limit their usage. Variable conditions in the MSC culture medium and the harvesting of exosomes, as well as the type of tumor, may affect the pro- or anti-tumor attributes of the MSC exosomes. These discrepancies in the features of MSC exosomes can vary the microenvironment of the tumor and the systemic environment of the host from tumor inhibition to tumor promotion, thereby affecting the outcome of treatment [37, 196]. Therefore, the achievement of favorable clinical effects with limited undesired toxicity requires further exploration in the field of engineering exosomes accompanied by advancement in the production and isolation of exosome techniques.

## Conclusions and prospects

Identifying exosomes, in particular MSC exosomes, as a cell-free material that produce inherently beneficial therapeutic effects offers a new paradigm for their application in cancer therapy and regenerative medicine. Exosomes maintain the therapeutic benefit of their origin cells without stimulating the pivotal concerns such as possible tumorigenesis and unwanted gene mutation of MSC therapies. Besides, the therapeutic effects of MSC exosomes may be improved by genetically modified MSC exosomes aiming to express special ligands that direct them toward a favorable target and transfer small molecular drugs directly to the target sites as a drug delivery mechanism. Correspondingly, various clinical trials have been designed and conducted or are ongoing to address their safety, feasibility, and efficacy in the human (Table3); however, various issues remain challenging. First, various techniques for the purification, isolation, and characterization of MSC exosomes used in different laboratory studies can lead to inconsistent results. Further, the MSCs commonly have a limited capacity to produce exosomes on a large scale required to exert beneficial clinical effects. Also, the characterization of MSC exosomes in various subpopulations and precise content characterization of the cargo must be investigated because of the existence of heterogeneity in exosome contents, which in turn, may modify the effect of the intervention on the target tissue [197]. Moreover, to investigate MSC-EV as a therapeutic agent, an appropriate method for evaluating their mechanism of biodistribution and targeting, therapeutic efficiency, and manner and function of MSC-EV in vivo should be used. Appropriate safety profiles, on the other hand, must be developed to assess the optimal therapeutic dose and potential side effects of repeated administration [198]. Further, the progress of an exosome isolation protocol that meets good manufacturing practice (GMP) standards is of paramount importance [197]. In sum, investigation of the therapeutic application of MSC exosomes is still in the early stages and the precise functional mechanism of exosomes remains largely unclear. Therefore, further experimental research is also needed to establish the optimal therapeutic dose, interval, suitable route, and isolation methods along with optimizing the culture conditions of exosomes for reaching favorable therapeutic outcomes with minimal untoward effects in patients suffering from various organ disorders or cancers. Future research must be directed toward the design of a conventional isolation system as well as the development of a new generation of engineered MSCs capable of producing an improved amount of exosomes capable of transferring various efficient safe molecules for the treatment of human disorders.

**Table 3:** Evaluation of MSC exosomes therapeutic potential in clinical trial registered in ClinicalTrials.gov (January 2021)

*Condition*	*Study phase*	*Participant number*	*Cell source*	*Cell type*	*Delivery route*	*Location*	*NCT number*
ARDS	1/2	169	n.a	Allogeneic	Inhalation	China	NCT04602104
COVID-19	1	24	AT	Allogeneic	Inhalation	China	NCT04276987
MODS	n.a	60	n.a	Allogeneic	IV	China	NCT04356300
Pulmonary infection	1/2	60	AT	Allogeneic	Inhalation	China	NCT04544215
AIS	1/2	5	n.a	Allogeneic	IP	Iran	NCT03384433
Dry eye	1/2	27	UC	Allogeneic	Inhalation	China	NCT04213248
Macular holes	1	44	n.a	Allogeneic	Intravitreal	China	NCT03437759
Preeclampsia	n.a	200	UC	Allogeneic	n.a	Egypt	NCT03562715
AD	1/2	9	AT	Allogeneic	Intranasal	China	NCT04388982

Note; *ARDS* acute respiratory distress syndrome, *COVID-19* coronavirus disease 2019, *MODS* multiple organ dysfunction syndromes, *AIS* acute ischemic stroke, *AD* Alzheimer's diseases, *AT* adipose tissue, *UC* umbilical cord, *IV* intravenous, *IP* intraperitoneal, *n.a* not available

## Data Availability

Not applicable.

## Abbreviations

MSCs: Mesenchymal stem/stromal cells
mRNA: Messenger RNA
miRNAs: MicroRNAs
EVs: Extracellular vesicles
MVBs: Multivesicular bodies
PDGF: Platelet-derived growth factor
FGF: Fibroblast growth factor
EGF: Epidermal growth factor
